# Benefits of ICU admission in critically ill patients: Whether instrumental variable methods or propensity scores should be used

**DOI:** 10.1186/1471-2288-11-132

**Published:** 2011-09-21

**Authors:** Romain Pirracchio, Charles Sprung, Didier Payen, Sylvie Chevret

**Affiliations:** 1 Département de Biostatistique et Informatique Médicale, Unité INSERM UMR 717, Hôpital Saint Louis, APHP; Université Paris 7 Diderot; 1 Avenue Claude Vellefaux, Paris, 75010, France; 2 Service d'Anesthésie Réanimation, Hôpital Européen Georges Pompidou, APHP; Université Paris V Descartes, Sorbonne Paris Cité; 20 rue Leblanc, Paris, 75015, France; 3 Département d'Anesthésie Réanimation SMUR, Hôpital Lariboisière, APHP; Université Paris 7 Diderot; 2 rue Ambroise Paré, Paris, 75010, France; 4 Department of Anesthesiology and Critical Care Medicine, Hadassah University Hospital, Ein-Karem; Kiryat Hadassah, Jerusalem, 91120, Israel

**Keywords:** Causal inference, Instrumental variables, Dichotomous outcome, Mortality, ICU

## Abstract

**Background:**

The assessment of the causal effect of Intensive Care Unit (ICU) admission generally involves usual observational designs and thus requires controlling for confounding variables. Instrumental variable analysis is an econometric technique that allows causal inferences of the effectiveness of some treatments during situations to be made when a randomized trial has not been or cannot be conducted. This technique relies on the existence of one variable or "instrument" that is supposed to achieve similar observations with a different treatment for "arbitrary" reasons, thus inducing substantial variation in the treatment decision with no direct effect on the outcome. The objective of the study was to assess the benefit in terms of hospital mortality of ICU admission in a cohort of patients proposed for ICU admission (ELDICUS cohort).

**Methods:**

Using this cohort of 8,201 patients triaged for ICU (including 6,752 (82.3%) patients admitted), the benefit of ICU admission was evaluated using 3 different approaches: instrumental variables, standard regression and propensity score matched analyses. We further evaluated the results obtained using different instrumental variable methods that have been proposed for dichotomous outcomes.

**Results:**

The physician's main specialization was found to be the best instrument. All instrumental variable models adequately reduced baseline imbalances, but failed to show a significant effect of ICU admission on hospital mortality, with confidence intervals far higher than those obtained in standard or propensity-based analyses.

**Conclusions:**

Instrumental variable methods offer an appealing alternative to handle the selection bias related to nonrandomized designs, especially when the presence of significant unmeasured confounding is suspected. Applied to the ELDICUS database, this analysis failed to show any significant beneficial effect of ICU admission on hospital mortality. This result could be due to the lack of statistical power of these methods.

## Background

Most studies on intensive care unit (ICU) triage have focused on either patients admitted to or rejected from the ICU [1-3]. Few studies have documented improved survival by comparing similar patients admitted to ICUs and regular departments. The limitation of such observational research is the nonrandom assignment of the treatments, which may lead to selection bias [4]. Concerning ICU care, confounding by severity could plausibly occur in either direction; patients' severity intrinsically influences both the triage decision and the outcome. Some physicians may have been concerned that individuals with more severe organ failures would not benefit from ICU care while other physicians might recommend ICU care as a 'last resort' for their sickest patients, for fear of unintended negative effects.

To provide causal evidence from observational data, notably in critical care [5], appropriate statistical tools have been proposed [6,7]. The propensity score (PS) was one of the first techniques that specifically addressed this question [6]. However, this method relies on the strong underlying assumption of exchangeability, that is, the absence of an unmeasured confounder, which cannot be tested. An attractive alternative approach is the instrumental variable (IV) method because it may consistently estimate the average treatment effect of exposure in marginal patients, even in the presence of unmeasured confounding. This method supposes that there is an instrument that is correlated with treatment but uncorrelated with unobserved patient severity. However, while PSs are mostly used in medical settings [8], IV has been the standard method in econometrics [9]. Although proposed in the Health Sciences setting [10], aside from introductory papers to IV for epidemiology [11,12], IV has been poorly and only recently applied in medical research [13-15].

In this paper, we sought to illustrate the use of the IV approach on an observational cohort study that aimed to evaluate the beneficial effect of ICU admission on hospital mortality (the ELDICUS study [16]). Our objective was to assess such a benefit by introducing the concept of IV and by reviewing and comparing different IV approaches with a special focus on the selection of a valid instrument and on the best regression method in the case of a dichotomous outcome. In addition, some comparison between PS and IV with regard to estimating the causal benefit of ICU care from a large observational database was also provided.

## Methods

### Data source

EDLICUS is a prospective cohort study that was conducted in seven European countries (France, Israel, Italy, Spain, United Kingdom, Netherland, and Denmark) from 1 September, 2003 until 1 March, 2005. All adult patients evaluated for ICU admission were included in the study. The primary objective was to evaluate the beneficial effect of ICU admission on mortality in the elderly.

### Study End Point and Covariates

The study end point was the in-hospital mortality.

Potential baseline confounding variables, such as age, gender, acute medical diagnosis and chronic disorders, and surgical status, were recorded, as were routinely used ICU scoring systems, namely, the Karnofsky performance status scale [17], which allows a global evaluation of the health status; the Glasgow Coma Score [18], which is evaluates the deepness of the coma; the SOFA score [19], which measures organ failures; and the SAPS II [20], which is a global evaluation of patient severity within the first 24 hours following ICU admission related to in-hospital mortality.

The country of enrolment and variables related to the physician responsible for the triage decision, namely, age, gender, main specialization, and years of ICU experience, were also recorded.

### Statistical Analysis

First, to provide some comparison, the beneficial effect of ICU admission on hospital mortality was estimated from a standard logistic model, unadjusted and adjusted to baseline covariates.

#### Propensity Score approach

A PS model to predict the probability that a given patient would be admitted to the ICU at his first triage, conditional on baseline-measured covariates, was obtained by fitting a multivariate logistic model [6]. Then, a matched-paired analysis was performed with callipers at 0.2 times the standard deviation of the logit of the estimated propensity score, as previously recommended [21]. The matching procedure was performed without replacement. The beneficial effect of ICU admission on hospital mortality was then estimated by fitting a logistic model applied to the propensity score matched database [22].

#### Instrumental Variable approach

This approach attempts to estimate causal effects by using differences in medical practice patterns as a quasi-experiment, bypassing the usual way that physicians allocate treatment according to prognosis and thus removing both measured and hidden sources of bias [23]. IV analysis begins with the identification of an IV that will be used in the first regression of a multiple-stage regression process.

##### Instrument selection

An IV is defined as an observable variable that is predictive of exposure but that has no direct effect on the outcome and that is independent of the unobserved confounders [24,12,25]. The potential IV should meet three requirements: (1) the IV must be uncorrelated with the outcome of interest, except through the effect of treatment (usually referred as the *main assumption*); (2) it must be highly predictive of the treatment (*strength of the IV*); (3) the relationship between the IV and the exposure must be unconfounded, i.e., the instrument should be unrelated to the patients' characteristics. Under these conditions, IV analysis provides an asymptotically unbiased estimate of the treatment effect on the outcome [26]. Because the *main assumption *is empirically unverifiable [27], the choice of the instrument should rely first on subject-matter knowledge, i.e., some arguments as to why the assumptions are reasonable. Data can then be used to test the plausibility of the IV assumptions.

After performing bibliographic research [28-31] and interviewing different experts in critical care and biostatistics, three potential IV were selected from the present database because they were considered (1) to influence the propensity to be admitted to the ICU, (2) not to influence patients' chances of surviving, except through ICU acceptance or refusal, and (3) not to be related to patients' characteristics. These three potential IV are as follows: the country of enrolment, physician's age (dichotomized into < or > 40 y/o) and specialization (dichotomized into anaesthesiologists *vs*. others). Concerning the country of enrolment, we dichotomized the variable "country of admission" into "low admission rate country" vs. "high admission rate country". The threshold admission rate used to classify the countries was set at 0.85, allowing us to divide the study sample into two groups of approximately equal size.

The choice of the best instrument was based on a two-step procedure. First, we explored the strength of the potential IV as evaluated by the partial F-statistic from the first-stage regression [27] and by the partial r^2^, the square of the partial correlation between the instrument and the treatment, conditional on other covariates in the model, as proposed by Bound et al. [32]. From the econometric literature, an F-statistic greater than 10 indicates that the instrument is not weak [23]. However, the computation of both r^2 ^and the F-statistic require transforming the treatment allocation into a continuous variable. To verify that such an approach to IV selection was also appropriate for binary variables, we also examined the ability of each potential IV to reduce the imbalance in the major covariates. To do so, we compared the mean standardized difference as stratified by the actual treatment with the mean standardized difference as stratified by the IV, as proposed by Rassen et al. [27]. According to these criteria, the best instrument was the variable associated with the highest F-statistics and partial r^2 ^and with the greater reduction in the mean standardized differences.

##### IV analysis

The most commonly used IV approach relies on linear models with two-stage least-squares (2SLS) [9]. The 2SLS estimator is named as such because it can be obtained by two consecutive ordinary least-squares (OLS) regressions. Similar to a propensity model, the first linear model aims to specify the relationship between treatment assignment, the instrument and potential confounding variables. One can then specify a model for the outcome that includes not the actual exposure but instead the exposure as estimated for the first-stage equation as well as the same set of confounding variables.

Let Y be the outcome of interest, X be the treatment, Z the instrument and *β *a measure of the effect of X on Y. When X and Z are binary variables, the classic IV estimator ${\overset{⌢}{\beta }}_{IV}$, also called the Wald estimator, can be written as follows:

$${\hat{\beta }}_{IV}=\frac{\hat{E}\left[Y|Z=1\right]-\hat{E}\left[Y|Z=0\right]}{\hat{E}\left[X|Z=1\right]-\hat{E}\left[X|Z=0\right]}$$

In the case of dichotomous outcomes, one cannot simply replace the second-stage of the 2SLS model with a logistic model [33]. To address this problem, other approaches have been proposed. Generalizations to nonlinear structural equations based on log-linear or probit modelling have been recommended [34,35]. Generalized methods of moments (GMM) estimation have been also proposed [36], but they have been shown to produce essentially the same results as the 2-stage logistic method [37]. However, all IV methods encounter problems in the presence of effect modification by unobserved confounders, and sensitivity analyses have generally been recommended [38,39].

Hence, after selecting the appropriate instrument, we applied and compared four IV approaches. Double stage least square [37] was applied first. The second IV approach was the double stage logistic regression [37] (2LR), in which the 2 linear models used in the 2SLS are replaced by two logistic regressions. Double stage probit structural equation models were also used [40]. Such probit models were specifically developed to derive probabilities and thus constrain the predicted values of exposure and outcome to the 0-1 range. However, unlike those of logistic models, the coefficients of probit models cannot be directly interpreted as the logarithms of odds ratios. To offer a more natural interpretation, it has been demonstrated that multiplying probit coefficients by 1.6 offers an acceptable approximation of the logistic coefficients [37]. Finally, we also used a three-stage model (3LS), as proposed by Angrist et al. [41]. Specifically, a logistic model was used to derive a predicted probability, which was then used as an instrument in a subsequent 2-stage least squares estimation procedure.

#### Parameters of interest

We initially used the odds ratio (OR); since this ratio is commonly used in the intensive care setting, its performance has been also widely studied in propensity-score methods [42], and it allowed for a comparison with the IV estimates derived from the 2LR and the probit models. However, ORs have been criticized and considered "not collapsible" [43]. It has been argued that both relative and absolute measures should be reported [44]. Therefore, we also estimated the risk differences (RD) by computing the difference between the proportions of non-ICU admitted subjects experiencing the outcome and the proportion of ICU admitted subjects experiencing the outcome, in the overall and in the propensity matched cohorts [45]. This analysis allowed for a comparison with the IV estimates derived from the 2LS and the 3LS models.

All statistical analyses were performed using R software packages http://www.R-project.org. Continuous variables are expressed as mean ± SD. Estimated ORs and RDs are given with their 95-per cent confidence intervals (95CI). We bootstrapped the standard errors for all IV estimators of treatment effects [46]. We used cluster sampling and conducted 1,000 iterations for bootstrapping.

## Results

A total of 8,201 patients were enrolled in the study: 6,752 (82.3%) patients were accepted, and 1,449 (17.7%) were rejected. Table 1 shows that major characteristics significantly differed between admitted and nonadmitted patients. The crude analysis revealed a reduction in hospital mortality associated with ICU admission (OR = 0.74, 95CI: 0.65-0.84, p < 0.0001; RD = -0.06, 95CI: -0.08;-0.03, p < 0.0001) (Tables 2 and 3). However, after adjusting for 35 baseline covariates considered associated with the outcome, ICU admission was associated with increased hospital mortality (OR = 1.25, 95CI: 1.07-1.46; p = 0.005; RD = 0.03, 95CI: 0.01; 0.05, p = 0.01).

**Table 1:** Selected baseline characteristics according to the triage decision

	Overall cohort			Propensity-based matched cohort		
	(n = 8,201)			(n = 2,762)		
	**ICU triage**			**ICU triage**		
				
	**Admitted**	**Refused**	**Standardized difference**	**Admitted**	**Refused**	**Standardized difference**
	**(n = 6,752)**	**(n = 1,449)**		**(n = 1,381)**	**(n = 1,381)**	

Age	59.41 ± 18.47	60.76 ± 17.41	**0.31**	63.15 ± 17.14	63.65 ± 17.50	**0.03**
SOFA	4.87 ± 2.93	4.71 ± 2.71	**-0.20**	4.54 ± 2.65	4.40 ± 2.60	**-0.05**
SAPS	30.30 ± 15.82	29.28 ± 14.91	**0.09**	31.88 ± 14.01	30.81 ± 14.55	**-0.07**
GCS	12.43 ± 4.30	12.93 ± 4.00	**0.17**	12.94 ± 3.78	13.14 ± 3.57	**0.05**
Karnofsky	79.17 ± 20.22	79.30 ± 18.95	**-0.35**	75.70 ± 21.03	74.57 ± 21.63	**-0.05**

(GCS: Glasgow Coma Scale)

**Table 2:** Effect of ICU admission on in-hospital mortality using standard logistic regression (crude and adjusted logistic models) and instrumental variable-based analyses (double-stage logistic regression and double-stage probit structural equation model)

	Odds Ratio	95CI	p-value
Crude Logistic Regression	0.74	0.65-0.84	< 0.01
Adjusted Logistic Regression	1.25	1.07-1.46	0.01
Propensity matched cohort	1.23	1.04-1.45	0.01
2LR	0.73	0.24-2.45	0.56
Probit Model	0.89	0.24-2.37	0.71

The association measure is the odds ratio (with 95% confidence interval, 95CI). 2LR: double stage logistic regression

**Table 3:** Effect of ICU admission on in-hospital mortality using standard linear regression (crude and adjusted ordinary least squares models) and instrumental variable-based analyses (double and triple stage least squares models)

	Risk Difference	95CI	p-value
Crude OLS	-0.06	-0.08--0.03	< 0.01
Adjusted OLS	0.03	0.01-0.05	0.01
Propensity matched cohort	0.04	0.01-0.08	< 0.01
2LS	0.01	-2.45-2.30	0.99
3LS	-0.05	-1.41-0.89	0.49

The association measure is the absolute mortality difference (with 95% confidence interval, 95CI). OLS: ordinary least squares, 2LS: double stage least squares, 3LS: triple stage least squares.

### Propensity Score Analysis

Propensity scores were derived from a nonparsimonious logistic model including 35 baseline covariates. Only 1,381 of the 6,752 (20.5%) patients could be matched to a nonadmitted patient, resulting in a matched population of 2,762 patients. The matching enabled us to reduce the mean standardized difference in baseline covariates (Table 1). Consistent with the adjusted analysis of the whole population, ICU admission was found to be associated with increased hospital mortality (OR = 1.23, 95CI: 1.04-1.45, p = 0.014; RD = 0.044, 95CI: 0.010; 0.078; p < 0.0001) (Tables 2 and 3).

### Instrumental Variable Analysis

#### Choice of the instrument

Three baseline variables were evaluated as potential instruments: country of enrolment, physician's age and physician's specialization. Table 4 summarizes the strength of these three potential instruments. According to the partial F-statistic and r^2 ^as well as on the estimated OR, the country of enrolment variable seemed to have the highest strength. However, examining the residual imbalance after stratification on the IV, the physician's age offered the most homogeneous reduction in the standardized differences in baseline risk factors. Considering the strength of the instrument and the reduction in the residual imbalance, the physician's specialization was the instrument that seemed to offer the best properties. The reduction in baseline imbalance using the physician's specialization was close to that achieved using the propensity score method.

**Table 4:** Evaluation of the qualities of the potential instruments

	OR [95CI]	Partial r^2^	Partial F-statistic	p-value	IV effect on ICU admission Estimate (SD)	Standardized Differences				
						
						Age	SOFA	SAPS	GCS	Karnofsky
Original cohort						0.311	-0.205	0.092	0.166	-0.352

**Instrumental Variable (IV)**										

Country of enrolment	2.90 [2.58-3.28]	0.006	54.90	< .0001	0.15 (0.01)	0.306	0.075	0.209	-0.001	-0.143
Physician's age	2.03 [1.81-2.27]	0.001	13.42	.0003	-0.13 (0.01)	-0.003	-0.036	0.042	-0.031	-0.013
Physician's main specialization	2.22 [1.96-2.51]	0.003	25.55	< .0001	0.10 (0.01)	-0.071	0.075	0.080	-0.094	0.048

Partial r^2^: square of the partial correlation between the instrument and the treatment. GCS: Glasgow Coma Scale. OR: odds ratio. 95CI: 95% confidence interval. The IV effect on ICU admission (denominator of the Wald estimator) is expressed as the estimate (SD) of the linear regression that models ICU acceptance according to the IV.

#### IV based estimation of treatment effect

Using the physician's specialization as an instrument, the various multistage approaches all yielded comparable point estimates.

Table 2 presents the OR for in-hospital death obtained by two different IV approaches: the double-stage logistic regression and the double stage probit structural equation model. Neither the logistic (OR: 0.73, 95CI: 0.24-2.45, p = 0.56) nor the probit model (OR: 0.89 95CI: 0.24-2.37, p = 0.71) found an effect of ICU admission on in-hospital mortality. However, the confidence intervals of the IV effects were far higher than those obtained with standard regression methods.

Table 3 presents the estimation of the RDs in hospital mortality between nonadmitted and admitted patients using the double and the triple stage least squares models approaches. Consistent with previous IV estimations, we found no effect of ICU admission on hospital mortality using the 2SLS method (RD: 0.005, 95CI: -2.45; 2.30, p = 0.99) or the triple-stage approach (RD: -0.05, 95CI: -1.41; 0.89, p = 0.49). Again, the confidence intervals of the IV estimators were far higher than those obtained with standard regression methods.

## Discussion

ELDICUS is an observational study that intended to assess the benefit of ICU admission on mortality. Most previous studies have been based on cohort data analysed by standard statistical methods [4]. However, because ICU admission is likely determined jointly with an individual's likelihood of death, conventional estimates might be biased [47,48]. The instrumental variable method, which was initially developed for use with econometrics, has been proposed to handle such sources of bias, but it is still seldom applied to medical data [26,13,15]. To our knowledge, this is the first study to use IV analysis to examine the effect of first ICU admission on in-hospital mortality on critically ill patients. We explored the results by IV methods, using different instruments and different methods adapted to dichotomous exposures and outcomes as sensitivity analyses [38]. These results were compared with those obtained by standard regressions and propensity based analyses, using the in-hospital mortality as the primary end point.

We first used PS matched analysis [49]. Both the adjusted and the propensity based analyses found ICU admission to be associated with increased hospital mortality. However, PS methods might have some limitations. First, given the large imbalance in sample sizes between admitted and nonadmitted patients (82.3% of patients admitted to the ICU), the matching-without-replacement approach resulted in a dramatic reduction in the sample size. Indeed, only 20.4% of admitted patients could be matched to nonadmitted patients. Second, the PS does not handle the situation of unmeasured confounding. In the context of critically ill patients, it is likely that all the prognostic factors for hospital mortality would not be measurable at the time of ICU triage. Therefore, we sought to compare the results obtained with the PS with those obtained with specific methods that would handle the potential unmeasured confounding.

Instrumental variable methods are becoming increasingly popular because they seem to overcome the problem of unobserved confounding in observational studies [25]. The principle of IV analysis is to evaluate how much the variation in the treatment variable that is induced by the instrument affects the outcome. Although appealing, IV methods rely on strong assumptions that might limit their use in practice: first, the absence of any direct effect of the instrument on the outcome (usually described as the *main *assumption); second, that the variation in the IV causes substantial variations in the treatment variable (usually described as the IV *strength*); and third, that the relationship between the IV and the treatment is unconfounded. The main issue is finding a good instrument. However, because these assumptions are not empirically verifiable [12,25] the choice of a *good instrument *first relies on carefully evaluating the key assumptions of IV when identifying a potential IV. In our example, three variables served as potential instruments. The first IV was countries of enrolment, which shared close populations in terms of health status and medical resources [31]. This IV found no effect on the outcome but did find variations in the treatment exposure due to the countries' own policies regarding ICU admission. The second IV, the physician's age, has been suggested to influence the triage decision [30] but not to modify the outcome, given that ICU care is not provided solely by the physician who admitted the patient. Finally, the third IV was the physician specialization, which was chosen because in most European countries ICU physicians may be anaesthesiologists or intensivists [29], and this characteristic may influence the admission policy while not affecting the outcome.

We then selected the best instrument from among these three potential IVs by examining the strength of the association between the IV and the treatment, as evaluated by the partial F-statistic and the partial r^2 ^from the first-stage regression [27,32]. All three selected instruments had partial F-statistics greater than 10, a threshold that supposedly indicates that the instrument is not weak [23]. However, the partial r^2 ^values were smaller than those usually reported in the medical or the economic literature [14,27]. Because the treatment variable was naturally binomial in our database, we sought to propose a more appropriate solution to evaluate the strength of the association between the IV and treatment. Using an OR as a measure of the association between treatment exposure and the IV, we found results similar to those obtained using the F-statistic or the partial r^2^. The quality of the instrument was also evaluated by its ability to reduce the imbalance in the major covariates [27]. However, the IV-based analysis yielded estimates far different from those obtained with the propensity-matched sample. Indeed, the propensity-based estimates were similar to those obtained with conventional multivariate regression models, supporting a negative effect of ICU admission on in-hospital mortality, while all IV analyses resulted in a lack of impact of ICU admission on in-hospital mortality. Of course, because we do not know the true association between ICU utilization and hospital death, we cannot formally conclude that the one method is better than the other. A simulation study to explore differences between these analytical methods with respect to controlling for confounding would be of interest. Nevertheless, in the context of ICU patients, because hospital mortality is usually considered highly multifactorial the presence of unmeasured confounders appears likely. The absence of concordance between PS- and IV-based estimates may support the existence of unmeasured confounding. However, as previously emphasized by several authors [32,23], the use of weak instruments may lead to large standard errors in the IV estimates or even bias in the IV estimates if the weakness is associated with a small sample size or a violation of the main assumption. In our case, IV methods undoubtedly yielded estimates with larger confidence intervals; thus, the limited partial r^2 ^can be considered a threat to the validity of the IV method. However, Martens et al. showed that when bias occurs in the IV estimates, it is in the direction of the ordinary least squares estimation [23]. In contrast, our results of the 2SLS estimator were far different from the results obtained using ordinary least square regression. This finding supports the idea that, despite the limited partial r^2 ^that may explain the large standard errors, the large sample size and the validity of the main assumption limited the bias in the IV estimates. Nevertheless, this finding could illustrate the low precision of the estimates and thus the low statistical power of treatment comparison.

The second limitation of IV techniques is that they rely on multiple stage linear models, which might be nonadaptive in the context of dichotomous outcome measures [37]. We compared the results obtained by the different methods previously proposed in the context of dichotomous outcomes [37] and found relatively large differences between the various IV approaches. Indeed, if all IV estimations led to a nonsignificant effect of ICU admission, then the 2SLS estimator was the only one that was far different from the crude analysis, which is expected to be the most biased method. As previously described in the case of weak instruments [23], all other IV estimators seemed biased in the direction of the unadjusted ordinary least squares estimation. Hence, our results strongly support the use of standard 2SLS methods, even when dealing with dichotomous outcome measures.

Our results could be compared with those based on a previously published propensity-based analysis of the ELDICUS database [16]. Our IV estimate did not conflict with previous PS estimates, though larger confidence intervals modified the conclusions. However, our PS results were different from those previously published [16]. This difference can be explained by major differences in the analytic procedure: first, we considered hospital mortality not 28- and 90-days mortalities; second, we used a PS matching method [21] whereas Iapichino provided estimates adjusted on PS quintiles. Thus, conditional estimates provided by Iapichino can substantially differ from marginal estimates reached by the former, especially when using the OR as the association measure, because of its noncollapsibility [43]. Moreover, we only assessed the benefit of the ICU first triage decision whereas Iapichino considered all the triages independently. Finally, differences in the patient selection should be stressed because we analysed a total of 8,201 patients including 6,752 (82%) first admissions. Conversely, Iapichino [16] included in the analysis of 28-day mortality 7,308 first admissions, a lower number because of the exclusion of patients with a lack of information on time of triage, triage decision, or outcome and the exclusion of those referred to a coronary unit. These results suggested an ICU benefit among severe patients and were confirmed with 6,500 patients triaged only once. It is likely that Iapichino's cohort included somewhat more severe patients, suggesting an ICU benefit among severe patients.

Finally, like randomized clinical trials, external validity depends on the studied population, and it should be emphasized that IV- and PS-matching attempt to estimate different effects of treatment. Indeed, IV approaches yield estimates of a local average treatment effect (LATE) [50-52] while propensity-based approaches yield estimates of the average treatment effect on the treated (ATT) [45]. Informally, the effect of ICU admission, as estimated via PS matching, can be defined as the effect observed in the patients admitted as compared with the effect observed in patients with a similar propensity for ICU admission but who were not admitted. PS matching does not capture the effect of ICU admission in nonadmitted patients who had a very low probability of being admitted. The IV approach yields estimates of the treatment effect not only in the treated but also in a restricted subgroup of patients for whom the instrument was informative about treatment assignment; these are the so-called "marginal" or *compliers*. *Noncompliers*, as opposed to *compliers*, are patients who, whatever the value of the instrument, would always have been treated or untreated. Hence, in our situation, the effect of ICU admission on hospital mortality is not captured by the IV approach for the patients who, whatever the value of the physician's specialization, i.e., the chosen instrument, would have always been accepted or rejected from the ICU. Thus, it is important for researchers to state the treatment-effect concept that they are trying to identify before beginning estimation [53].

## Conclusion

Instrumental variable methods offer an appealing alternative to handle the selection bias related to nonrandomized designs, especially when the presence of significant unmeasured confounding is suspected. Applied to the ELDICUS database, this analysis failed to show any significant beneficial effect of ICU admission on hospital mortality. When the clinical question underlying the creation of the database is to assess a local average treatment effect, effort should be made to incorporate in the dataset covariates that behave as appropriate instruments, allowing IV analysis if the presence of unmeasured confounding is suspected.

## List of Abbreviations

IV: instrumental variables; PS: propensity score; ICU: intensive care unit; GMM: Generalized methods of moments; OR: odds ratio; RD: risk difference; 95CI: 95% Confidence interval; LATE: local average treatment effect; ATT: average treatment effect on the treated

## Competing interests

The authors declare that they have no competing interests.

## Authors' contributions

RP performed the analysis and wrote the manuscript, CS was the principal investigator of ELDICUS, DP was the French principal investigator of ELDICUS, and SC supervised the analysis and the elaboration of the manuscript. All authors read and approved the final manuscript.

## Pre-publication history

The pre-publication history for this paper can be accessed here:

http://www.biomedcentral.com/1471-2288/11/132/prepub
